# ﻿Taxonomic studies on the genus *Sanicula* (Apiaceae) from China (III): The morphology and distribution of *S.caerulescens* (Apiaceae), with *S.oviformis* reduced to a new synonym

**DOI:** 10.3897/phytokeys.249.136854

**Published:** 2024-11-20

**Authors:** Hui-min Li, Wei Zhou, Jun-wen Zhu, Chun-feng Song

**Affiliations:** 1 Jiangsu Key Laboratory for the Research and Utilization of Plant Resources, Institute of Botany, Jiangsu Province and Chinese Academy of Sciences (Nanjing Botanical Garden Mem. Sun Yat-Sen), Nanjing 210014, Jiangsu, China Institute of Botany, Jiangsu Province and Chinese Academy of Sciences Nanjing China

**Keywords:** Chongqing, morphology, *Sanicula*, taxonomy, Umbelliferae

## Abstract

In the present study, we examined the morphological variations within *Saniculacaerulescens* and determined the identity of *S.oviformis* through observations of herbarium specimens (including type material) and field studies of plants in their type locality. Our findings revealed that *S.oviformis*, originally described from Nanchuan County in southern Chongqing, is conspecific with *S.caerulescens*. This species is mainly distributed in Chongqing, Guizhou, western Hunan, Sichuan and north-eastern Yunnan in China, as well as in Hà Giang in Vietnam. Key morphological characters of leaves, inflorescences and fruits confirm this synonymy. Based on these findings, we propose reducing *S.oviformis* to a synonym of *S.caerulescens*. Additionally, the geographical distribution of *S.caerulescens* is clarified.

## ﻿Introduction

**Figure 1.**
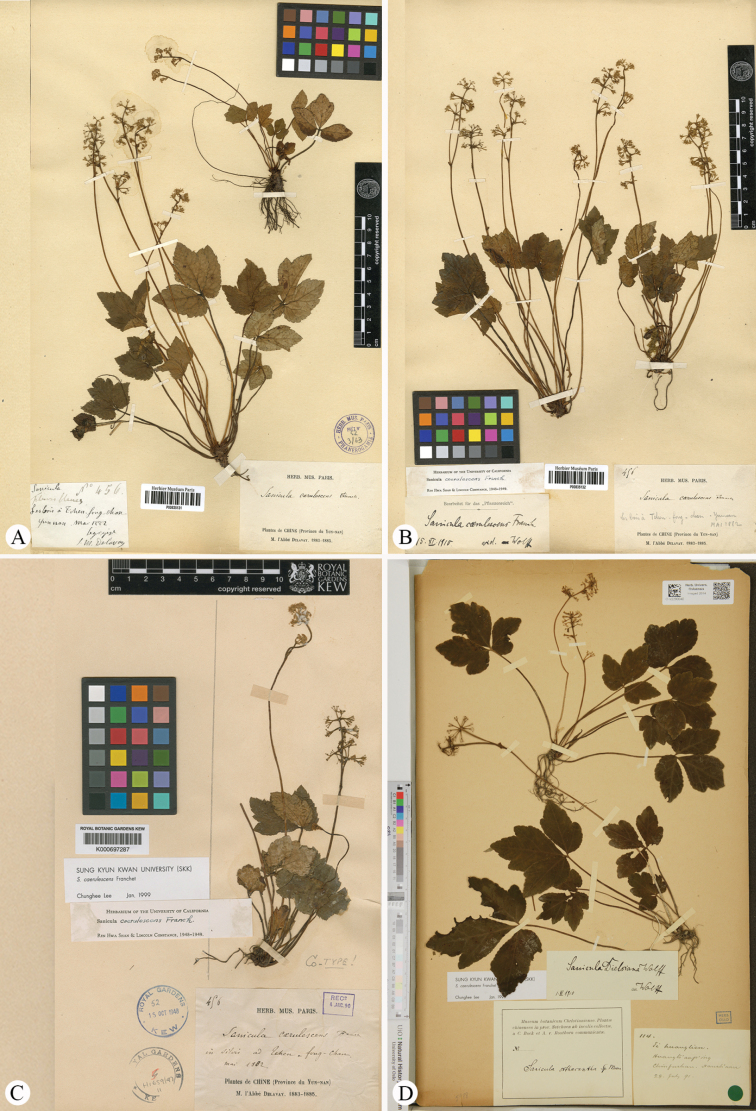
Lectotype (**A**) and isolectotype (**B**, **C**) sheets of *Saniculacaerulescens*, alongside the holotype (**D**) sheet of *S.dielsiana*.

*Saniculacaerulescens* Franchet was described on the basis of one gathering, *M. Delavay 456* (K000697287, P00835131, P00835132; Fig. 1A–C), from Chengfeng Shan (also referred to Tchen-fong-chan) in Shuifu, Zhaotong City, located in north-eastern Yunnan Province, southwest China. In the protologue, Franchet (1894) emphasised two distinguishing features of *S.caerulescens* in comparison with *S.orthacantha* Moore – foliar morphology and arrangement of its inflorescences. Specifically, the leaves of *S.caerulescens* have petiolate segments, with lateral segments being either entire or slightly lobed. The inflorescences of this species are characterised by a floral branch that may be either simple or irregularly bipartite, with each arrangement supporting three or four sessile umbels. Additionally, the lower peduncle of the floral branch, as well as the sessile umbels, is typically accompanied by a small leaf, which lacks the subulate bracts that are uniquely present at the base of the pedicels. This particular inflorescence structure is markedly different from that of *S.orthacantha*.

Shan and Constance (1951) recognised this species and noted that its original dichasial branching has been lost, leading to the formation of a pseudoracemose inflorescence. They subsequently reduced *S.dielsiana* Wolff and *S.stapfiana* Wolff as synonyms of *S.caerulescens*. Hiroe (1979) accepted their treatment.

Since its original description, *S.caerulescens* has consistently been regarded as a distinct species by numerous authors, including Diels (1901), Boissieu (1906), Wolff (1913), Shan and Constance (1951), Hiroe (1979), Liou (1979), Wu (1984), Yang (1989), Liu (1997), Sheh and Phillippe (2005) and Pimenov (2017). The species has been recorded in south-western China (Chongqing, Guizhou, Hunan, Sichuan and Yunnan) and north-eastern Vietnam (Liou 1979; Wu 1984; Yang 1989; Liu 1997; Sheh and Phillippe 2005; Sun 2010; Pimenov 2017).

**Figure 2.**
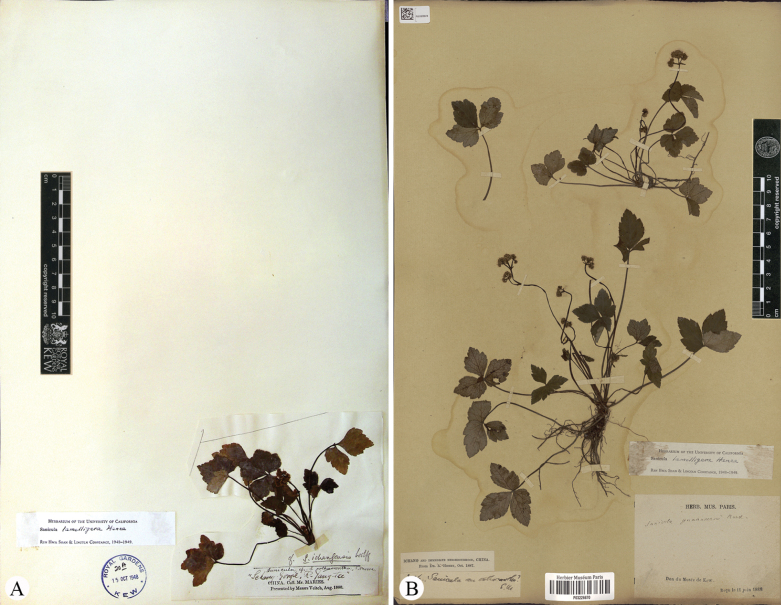
Specimens (**A, B**) of *Saniculalamelligera* from Ichang and neighbouring areas in Hubei Province, all previously misidentified and cited by Diels (1901) as *S.orthacantha* and by Wolff (1913) as *S.dielsiana***A***C. Maries s.n.* (K) **B***A. Henry 3526* (P).

**Figure 3.**
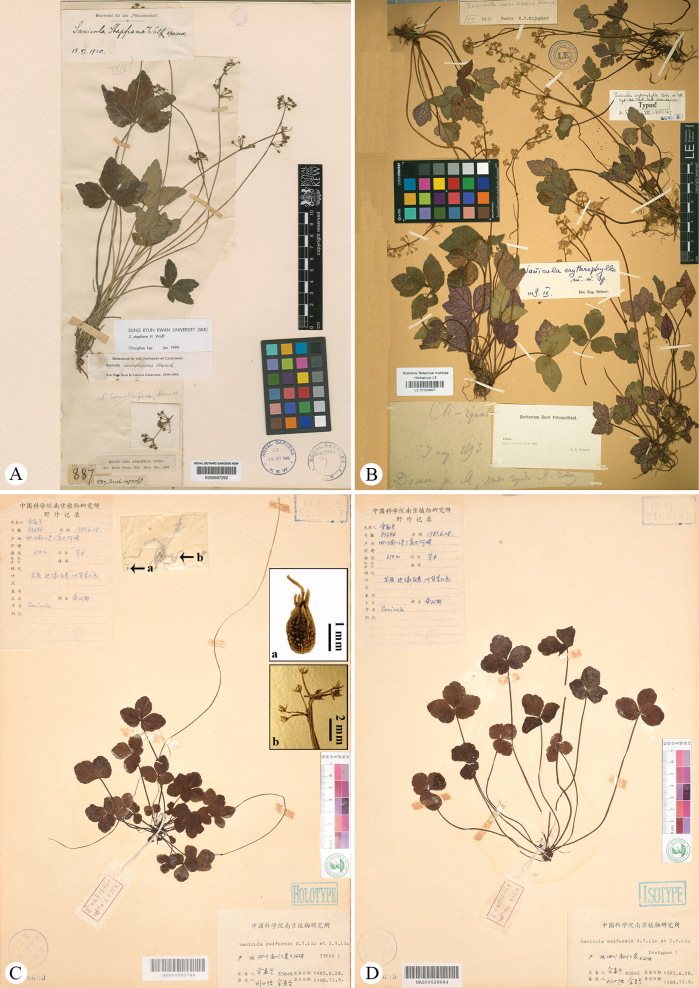
Holotype (**A**) sheet of *Saniculastapfiana*, holotype (**B**) sheet of *S.erythrophylla* and holotype (**C**) and isotype (**D**) sheets of *S.oviformis*.

*Saniculadielsiana* Wolff was described based on a single specimen, *C. Bock & A.V. Rosthorn 114* (O-V2290040; Fig. 1D), collected from Mount Jinfo (also referred to as Chin fu shan) in Nanchuan County, located in southern Chongqing Municipality (a directly-administered municipality formerly belonging to Sichuan Province) (Wolff 1910). Later, Wolff (1913) recognised this species and noted that Diels (1901) had initially cited its three collections under *S.orthacantha*. These collections included *C. Bock & A. v. Rosthorn 114* (Fig. 1D), along with two additional specimens: *C. Maries s.n.* (K; Fig. 2A) from Ichang and *A. Henry 3526* (P03226670, US03074343; Fig. 2B; only P03226670 is shown here) from Ichang and its surrounding areas in Hubei Province. However, our preliminary examination indicated that the latter two collections were misidentified and actually belong to *S.lamelligera*. Meanwhile, Wolff (1913) described *S.stapfiana* Wolff, based on *E. Faber 887* (K000697292, MO215722; Fig. 3A; only K000697292 is shown here) from Mount Emei (also known as Mount Omei) in south-western Sichuan. Shan and Constance (1951) later concluded that both *S.dielsiana* and *S.stapfiana* were described from the same region and likely represent minor variants of *S.caerulescens*. Thus, they treated both as synonyms of *S.caerulescens*. This treatment has been accepted by Hiroe (1979), Liou (1979), Liu (1997), Sheh and Phillippe (2005) and Pimenov (2017).

*Saniculaerythrophylla* Bobrov was described based on a single collection, *G.N. Potanin s.n.* (LE01029607; Fig. 3B) from the Ya River Valley, situated below the City of Ya’an (also known as Ya Chou) in western Sichuan (Bobrov 1950). In the protologue, Bobrov highlighted two features that set this species apart from other members of S.ser.Orthacantha Wolff – its aphyllous stems and the lilac-purple undersides of its tripartite leaves. Despite this detailed description, the species received little attention in subsequent taxonomic treatments until Liou (1979) synonymised it with *S.caerulescens* without offering a rationale. This synonymisation was later endorsed by Liu (1997), Sheh and Phillippe (2005) and Pimenov (2017). The latter author also designated the P00835131 sheet as the lectotype of *S.caerulescens* (Fig. 1A).

*Saniculaoviformis* X.T. Liu & Z.Y. Liu was described from a single collection, *M.L. Sheh 83646* (NAS00028684, NAS00028685, NAS00082799; Fig. 3C, D; NAS00028684 and NAS00082799 are shown here) from Mount Jinfo (Sheh et al. 1991). In the protologue, the authors indicated that *S.oviformis* is closely related to *S.lamelligera* Hance, sharing traits such as generally long and thin stems (vs. short and erect), palmately trisect leaves with ovate or obovate segments and slightly crenate or nearly entire margins (vs. medium segments slightly trisect, lateral segments bipartite or lobate, with erose serrate margins) and fruit spines lacking base lamellae (vs. spines with base lamellae). Although only known from the type material, *S.oviformis* has been consistently recognised as a distinct, endemic species restricted to its type locality by subsequent authors, including Sheh and Phillippe (2005) and Pimenov (2017).

The aim of this study is to elucidate the morphological variation of *S.caerulescens* and to determine the identity of *S.oviformis*, based on observations of herbarium specimens (including type material) and living plants in the field.

## ﻿Materials and methods

For morphological comparisons, we conducted a thorough examination of specimens or high-resolution images of related *Sanicula* L. from the following herbaria: A, AU, BM, CDBI, CDCM, CSFI, CSH, E, FJIDC, FJSI, GNUG, GXMG, GXMI, GYBG, GZAC, GZTM, HAST, HGAS, HHE, HUFD, HX, HZ, IBK, JIU, JJF, JSPC, K, KUN, L, LBG, LE, MO, N, NAS, NTUF, NY, O, P, PE, PEY, QNUN, SM, SYS, SZG, TAI, TAIF, TI, TIE, US, WCSBG, WUK, ZM and ZY. Field observations were conducted across six populations in Chongqing, Hubei, Sichuan and Yunnan Provinces. Of these, four key populations are highlighted: one from Mount Jinfo in Nanchuan County, southern Chongqing, the type locality of *S.dielsiana* and *S.oviformis*; one from Shizhu County in south-eastern Chongqing; one from Mount Emei in south-western Sichuan, the type locality of *S.stapfiana*; and one from Yongshan County in Zhaotong, north-eastern Yunnan, the type locality of *S.caerulescens*. The morphological comparisons presented are the result of a comprehensive analysis of both herbarium specimens and fresh materials collected during our fieldwork.

## ﻿Results and discussion

**Figure 4.**
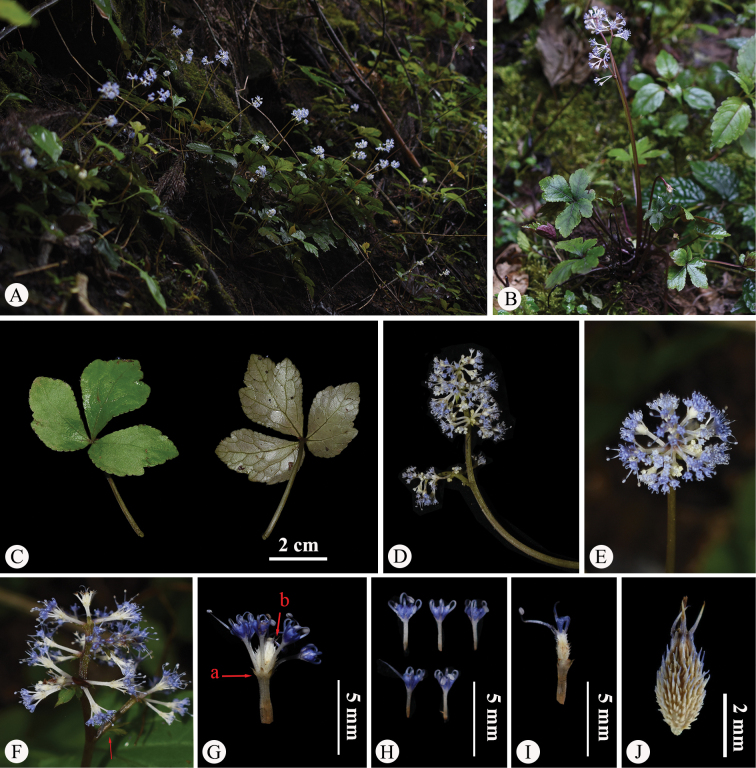
*Saniculacaerulescens* in the wild (China, Yunnan, Zhaotong, the type locality of *S.caerulescens*) **A** habitat **B** habit **C** leaf (left: adaxial surface; right: abaxial surface) **D** portion of inflorescence (side view) **E** portion of inflorescence (top view) **F** portion of inflorescence, with the arrow indicating upper reduced and sheathing cauline leaves; **G** umbellule (side view; **a** involucellate bracteoles, **b** calyx teeth) **H** staminate flowers (side view) **I** fertile flower with fruit, stamens, petals and calyx teeth **J** mericarps. Photographed by Hui-Min Li.

The type material of *Saniculacaerulescens* (Fig. 1A–C) has 2–7 stems, approximately 10–30 cm long. The basal leaves are numerous, cordate-ovate in shape and vary in texture from chartaceous to subcoriaceous. These leaves follow a subternate to trifoliate pattern, with distinctly petiolulate segments. The median segment is obovate to ovate-lanceolate, measuring 1.0–4.0 cm in length and 0.7–3.0 cm in width, with a cuneate base tapering into a petiolule and an obtusely trilobed apex. The lateral segments are obliquely ovate, shallowly bilobed and feature crenate-serrate margins with spinose teeth. All segments are supported by petioles several times longer than the leaf blades. The upper cauline leaves are considerably reduced, forming small sheaths 2.0–5.0 mm long, with pinnately lobed blades. The inflorescence has a pseudoracemose structure, with lower umbels either sessile or borne on short peduncles along the main branches of the stem. The fruits are covered with short spicules, which are either erect or slightly curved. These spicules occasionally feature membranous bases or remain underdeveloped along the fruit’s furrows. These morphological features were corroborated through field observations of living plants in Yongshan County, Zhaotong, north-eastern Yunnan (Fig. 4), the type locality of *S.caerulescens*. Furthermore, the lectotype designated by Pimenov (2017), i.e. *M. Delavay 456* (P00835131; Fig. 1A), aligned with the original Latin description precisely and is well-preserved, reaffirming the validity of the lectotypification, which we hereby accept.

**Figure 5.**
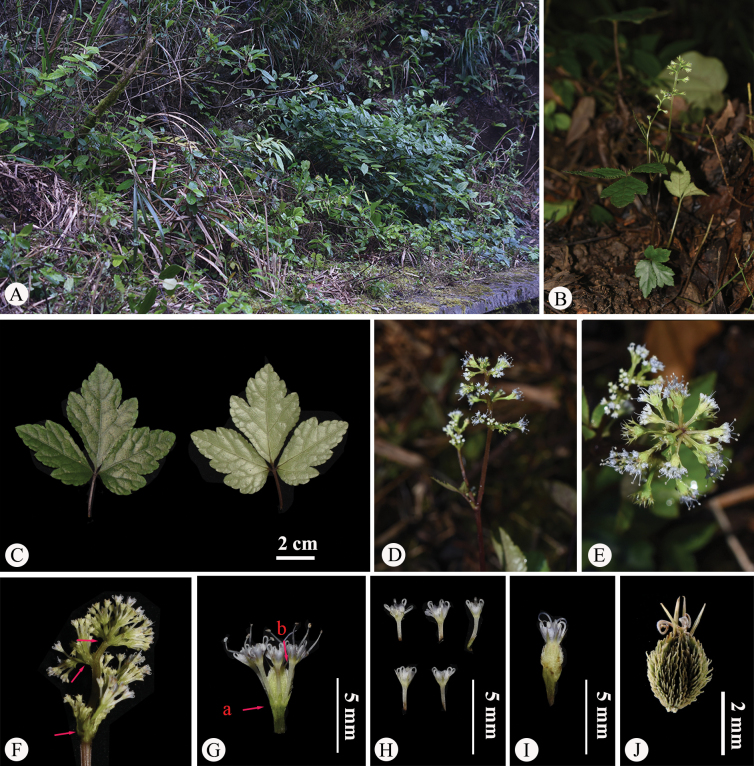
*Saniculacaerulescens* in the wild (China, Chongqing, Nanchuan, Mount Jinfo, the type locality of *S.dielsiana* and *S.oviformis*) **A** habitat **B** habit **C** leaf (left: adaxial surface; right: abaxial surface) **D** portion of inflorescence (side view) **E** portion of inflorescence (top view) **F** portion of inflorescence, with the arrow indicating upper reduced and sheathing cauline leaves **G** umbellule (side view; **a** involucellate bracteoles, **b** calyx teeth) **H** staminate flowers (side view) **I** fertile flower with fruit, stamens, style, petals and calyx teeth **J** mericarps. Photographed by Hui-Min Li.

The type specimens of *Saniculadielsiana* (Fig. 1D) have 1–2 stems 12–30 cm long. The basal and upper cauline leaves are similar to those of *S.caerulescens*, characterised by slight and continuous variations in both length and width. The median segments of the basal leaves measure 2.0–6.5 cm in length and 2.0–5.0 cm in width, whereas the upper cauline leaves range from 2.0 to 8.0 mm in length. No significant differences were noted in the characters of the inflorescence or fruit. These observations were further supported by field studies conducted at the type locality, Mount Jinfo, located in Nanchuan County, southern Chongqing (Fig. 5).

**Figure 6.**
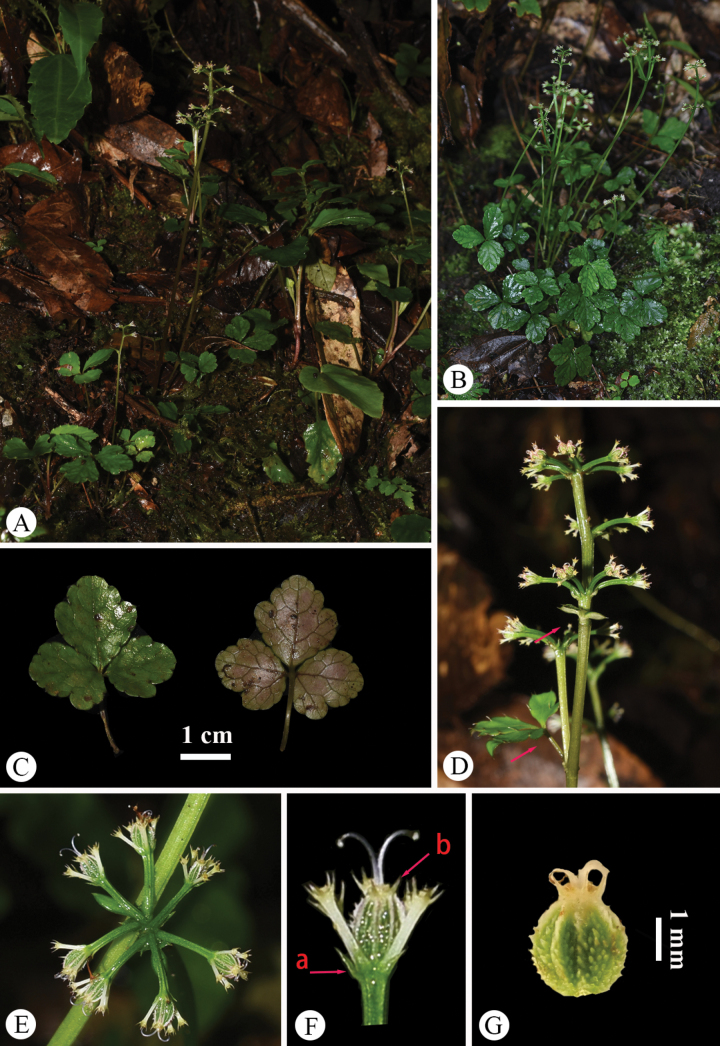
*Saniculacaerulescens* in the wild (China, Chongqing, Shizhu) **A** habitat **B** habit **C** leaf (left: adaxial surface; right: abaxial surface) **D** portion of inflorescence (side view), with the arrow indicating upper reduced and sheathing cauline leaves **E** portion of inflorescence (top view) **F** umbellule (side view; **a** involucellate bracteoles, **b** calyx teeth) **G** fertile flower with fruit, style and calyx teeth. Photographed by Hui-Min Li.

*Saniculaoviformis*, described from the same locality as *S.dielsiana* (Mount Jinfo, Nanchuan County, southern Chongqing), was found to have been based on a rather depauperate type specimen with a significantly damaged inflorescence and flowers. Amongst the examined sheets, only the holotype sheet NAS00082799 has a pseudoracemose inflorescence, with the lower umbels being either sessile or short-pedunculate and the fruits covered with spicules (Fig. 3C). The stem length is approximately 30 cm. The basal leaves are numerous, cordate-ovate in shape and have a subcoriaceous texture. These leaves follow a trifoliate pattern, with distinctly petiolulate segments. The median segment is broadly ovate, measuring 0.8–3.0 cm in length and 0.6–2.0 cm in width, with a broadly cuneate base narrowing into a petiolule and an obtusely trilobed apex. The lateral segments are obliquely ovate, shallowly bilobed and feature crenate-serrate margins with spinose teeth. All segments are supported by petioles that are several times longer than the leaf blades. The upper cauline leaves are significantly reduced, forming small sheaths measuring 1.5–4.0 mm in length, with pinnately lobed blades. Although the type specimens reveal that *S.oviformis* has relatively diminutive leaves, our discovery of a population in Shizhu County, south-eastern Chongqing, near Nanchuan County (Fig. 6), demonstrated a significant variability in leaf size both within and between populations. Thus, the small leaves of *S.oviformis* were considered to exhibit a considerable morphological variability.

**Figure 7.**
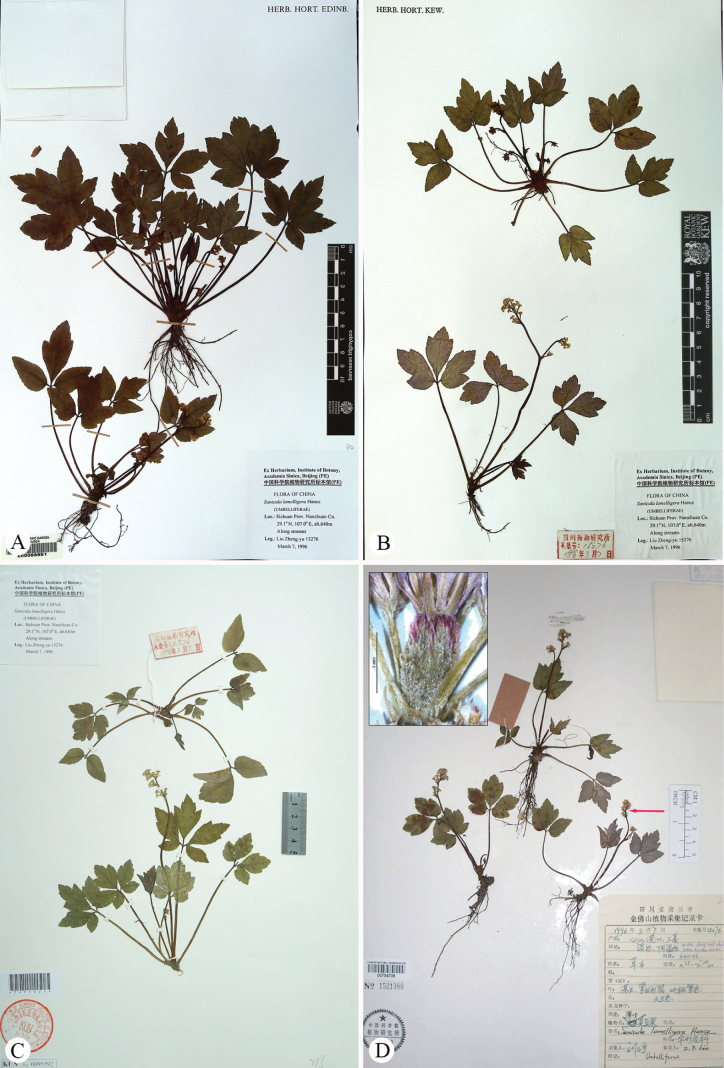
Specimens of *Saniculacaerulescens* collected from Chongqing, Nanchuan. All misidentified as *S.lamelligera* by Z.Y. Liu, a co-author of *S.oviformis***A***Z.Y. Liu 15276* (E) **B***Z.Y. Liu 15276* (K) **C***Z.Y. Liu 15276* (KUN) **D***Z.Y. Liu 15276* (PE; the arrow indicating the spinous fruit).

**Figure 8.**
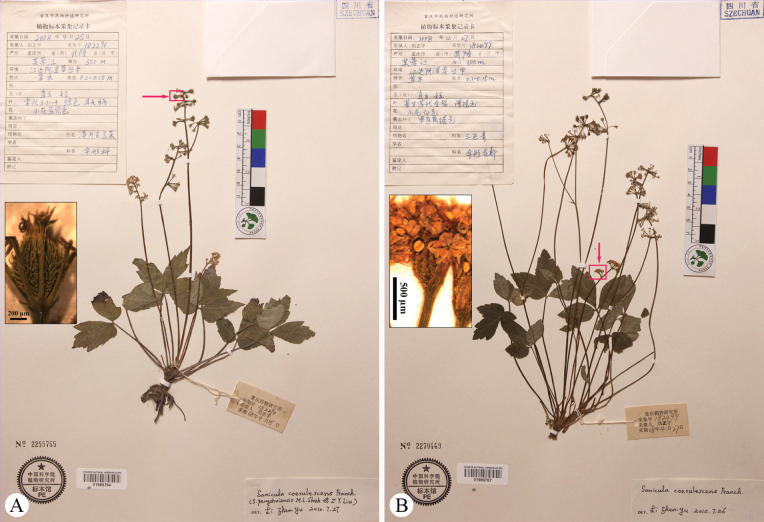
Specimens of *Saniculacaerulescens* collected from Chongqing, Wulong **A***Z.Y. Liu 182290* (PE) **B***Z.Y. Liu 182499* (PE). All arrows indicating the spinous fruits.

As previously noted, *S.oviformis* was compared with *S.lamelligera* by Sheh et al. (1991), with the latter distinguished by a range of morphological characters. In a subsequent study, we conducted a more detailed examination of *S.lamelligera* (Li and Song 2022). Notably, the sheets of *Z.Y. Liu 15276* (E00088651, HAST070536, K, KUN0465625, P03226696, PE00754708; Fig. 7; only E00088651, K, KUN0465625, and PE00754708 are shown here) from Mount Jinfo, Nanchuan, Chongqing Municipality, were misidentified as *S.lamelligera* by Z.Y. Liu, a co-author of *S.oviformis*. Additionally, two gatherings collected along the Furong River in Wulong County (a district neighbouring Nanchuan) were labelled by Liu as distinct species on their determination slips, i.e. *Z.Y. Liu 182290* (PE01989764; Fig. 8A) and *Z.Y. Liu 182499* (PE1989767; Fig. 8B). The former was labelled as either *S.caerulescens* or *S.pengshuiensis* – which, according to Li and Song (2022), should now be treated under *S.lamelligera*. We identified the sheet PE1989767 as *S.caerulescens*. These discrepancies suggest that the authors may not have fully grasped the extent of morphological variability within *S.lamelligera* or *S.caerulescens*. A detailed morphological comparison between the two species is provided in Table 1. Additionally, it is noteworthy that no additional specimens of *S.oviformis* have been recorded since its original description.

**Table 1.** Morphological comparisons between *Saniculalamelligera* and *S.caerulescens*.

	***S.lamelligera***	***S.caerulescens***
Roots	rhizome short, woody, bearing fibrous roots, occasionally with nodes	rhizome, rarely stoloniferous, oblique rootstock, bearing fibrous roots
Stems	slender and erect, few branches above the middle, 8–40 cm tall	slender and erect, several branches above the middle, 5–40 cm tall
Basal leaves	cordate suborbicular, ternate, segments distinctly petiolate; median segments cuneate-obovate to rhombic, obtuse to acute and more or less trilobed at the apex; lateral segments oblique, bilobed to the middle or only notched or rarely entire; margins crenate-serrate with spinose teeth.	cordate-ovate or ovate, sub-ternate to trifoliate; median segments obovate to ovate-lanceolate, distinctly shallowly trilobed, petiolate; lateral segments obliquely ovate, shallowly bilobed; margins irregularly crenulate-serrate with spinose teeth
Cauline leaves	reduced, trisect or undivided, bract-like, linear-lanceolate to obovate-lanceolate, ca. 5 mm long, shortly petiolate or sessile	significantly reduced, degenerating into sheathing, trisect or undivided, bract-like, ca. 2.0 mm long, subsessile or sessile
Inflorescence	1- or 2- to several branches	pseudo-racemose, sometimes the lower umbels in fascicles, sessile or short-pedunculate
Involucrate bracts	trisect or linear-lanceolate, ca. 2 mm long	lanceolate to ovate, acuminate, ca. 1.6 mm long
Rays of umbels	1–7, ca. 3.5 mm long	terminal umbels pedunculate, 3- to 12-radiate, 2.5–7.0 mm long
Involucellate bracteoles	4–6, linear to lanceolate, ca. 0.6 mm long	4–6(–8), linear to lanceolate, ca. 1 mm long
Umbellules	4–7-flowered	5–7-flowered
Staminate flower	3–6 per umbellule; pedicels ca. 1.5 mm long; petals white, pinkish or bluish-purple	4–6 per umbellule; pedicels 2.1–4.0 mm long; petals mainly blue to purple
Fertile flower	1 per umbellule, sessile	1 per umbellule, sessile
Mericarps	variably spiculate-lamellate, comb-like	covered with short and straight spinous bristles, usually fused at the base, forming a thin tier
Vittae	5	5

**Figure 9.**
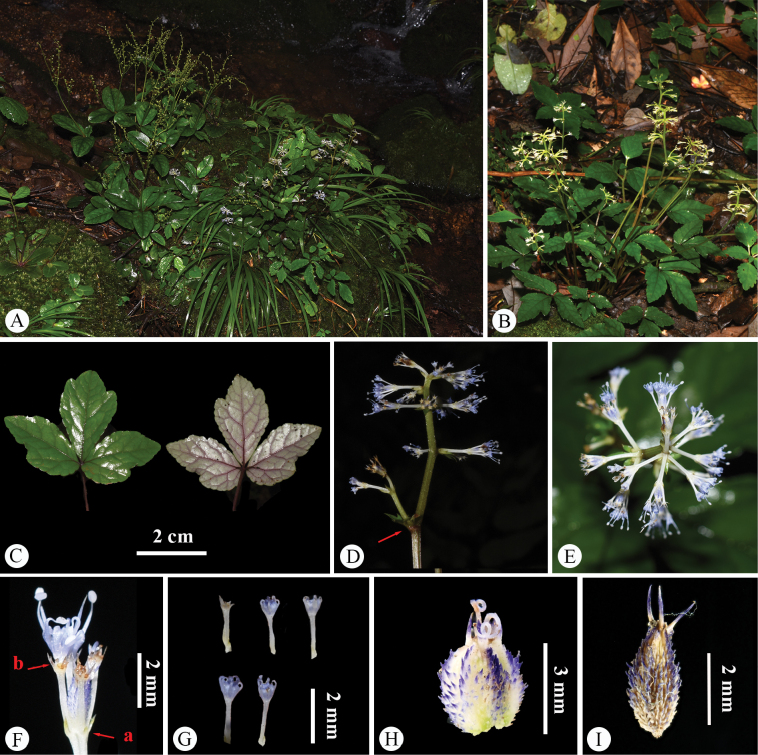
*Saniculacaerulescens* in wild (China, Emei Shan, the type locality of *S.stapfiana*) **A** habitat **B** habit **C** leaf (left: adaxial surface; right: abaxial surface) **D** portion of inflorescence (side view), with the arrow indicating upper reduced and sheathing cauline leaves **E** portion of inflorescence (top view) **F** umbellule (side view; **a** involucellate bracteoles, **b** calyx teeth) **G** staminate flowers (side view) **H** fertile flower with fruit, style and calyx teeth **I** mericarps. Photographed by Hui-Min Li.

In our examination of the type specimens of *Saniculastapfiana* (Fig. 3A) and *S.erythrophylla* (Fig. 3B), particularly concerning critical diagnostic characters, such as leaves, inflorescence and fruits, no significant differences were observed when compared to *S.caerulescens*. These observations were further corroborated by field studies conducted at Mount Emei, the type locality for *S.stapfiana*, located in south-western Sichuan (Fig. 9).

In conclusion, *Saniculadielsiana*, *S.erythrophylla*, *S.stapfiana* and *S.oviformis* are morphologically indistinguishable from *S.caerulescens*. Therefore, we endorse the treatments by Shan and Constance (1951) and Liou (1979), reducing *S.dielsiana*, *S.stapfiana* and *S.erythrophylla* to synonyms of *S.caerulescens*. Additionally, we also propose to synonymise *S.oviformis* with *S.caerulescens*.

### ﻿Taxonomic treatments

#### ﻿ Sanicula caerulescens

Taxon classificationPlantaeApialesApiaceae

Franch., Bull. Soc. Philom. Paris 8 (6): 109. 1984.

E707F9B0-9469-5390-B483-16A3DDFF1463

Figs 1 , 3 , 4 , 5 , 6 , 7 , 8 , 9

= Saniculadielsiana Wolff, Repert. Sp. Nov. Regni Veg. 8: 524. 1910. Type: China. Chongqing, Nanchuan, Mount Jinfo (= Chin fu shan), 28 July 1891, *C. Bock & A.V. Rosthorn 114* (holotype: O-V2290040!).
= Saniculastapfiana Wolff in Engler, Pflanzenr. 4 (228): 58. 1913. Type: China. Sichuan, Mount Emei (= Omei), alt. 3000 ft = 915 m, December 1887, *E. Faber 887* (holotype: K000697292!; isotype: MO215722!).
= Saniculaerythrophylla Bobrov, Bot. Mater. Gerb. Bot. Inst. Komarova Akad. Nauk S.S.S.R. 13: 167. 1950. Type: China. Sichuan, Ya’an (= Ya Chou), the valley of Ya River, 4 April 1893, *G.N. Potanin s.n.* (holotype: LE01029607!).
= Saniculaoviformis X.T. Liu & Z.Y. Liu, Acta Phytotax. Sin. 29 (5): 471. 1991, syn. nov. Type: China. Chongqing, Nanchuan, Mount Jinfo, alt. 650 m, 28 June 1983, *M.L. Sheh 83646* (holotype: NAS00082799!; isotypes: NAS00028684!, NAS00028685!).

##### Type.

China. Yunnan, Zhaotong, Shuifu, Chengfeng Shan (= Tchen-fong-chan), May 1882, *M. Delavay 456* [lectotype: P00835131! designated by Pimenov (2017); isolectotypes: K000697287!, P00835132!]. Fig. 1A–C.

##### Description.

Perennial. Rhizome, rarely stoloniferous, oblique rootstock, bearing fibrous roots. Stems 2–7, slender and erect, several branches above the middle, 5–40 cm tall. Basal leaves 2–14(–23), long petiolate; petioles 2–17 cm long; blade glabrous adaxially and abaxially, usually purplish-red on the back, 1–7 cm long, (2.2–)4.5–14 cm wide, cordate-ovate or ovate, sub-ternate to trifoliate, the median segment obovate to ovate-lanceolate, distinctly shallowly trilobed, petiolate, the lateral segments obliquely ovate, shallowly bilobed, the margins irregularly crenulate-serrate with spinose teeth. Cauline leaves significantly reduced, sheathing, subsessile or sessile, with pinnately lobed blades, resembling involucrate bract, ca. 2.0 mm long. Inflorescence pseudo-racemose, sometimes the lower umbels in fascicles, sessile or short-pedunculate; involucrate bract lanceolate to ovate, acuminate, ca. 1.6 mm long; terminal umbels pedunculate, 3- to 12-radiate, 2.5–7.0 mm long; involucellate bracteoles 4–6(–8), linear to lanceolate, ca. 1 mm long. Umbellules 5–7-flowered, staminate flowers 4–6 per umbellule, pedicels 2.1–4.0 mm long, petals mainly blue to purple. Fertile flowers 1 per umbellule, sessile; calyx teeth linear to lanceolate, acute, ca. 0.8 mm long; styles ca. 2.0 mm long, recurved. Mericarps globose to ellipsoid, 2.0–3.0 mm long, 1.0–1.5 mm broad, covered with short and straight spinous bristles usually fused at the base forming a thin tier; mericarp flattened dorsally, orbiculate in cross-section. Vittae 5.

##### Distribution.

**Figure 10.**
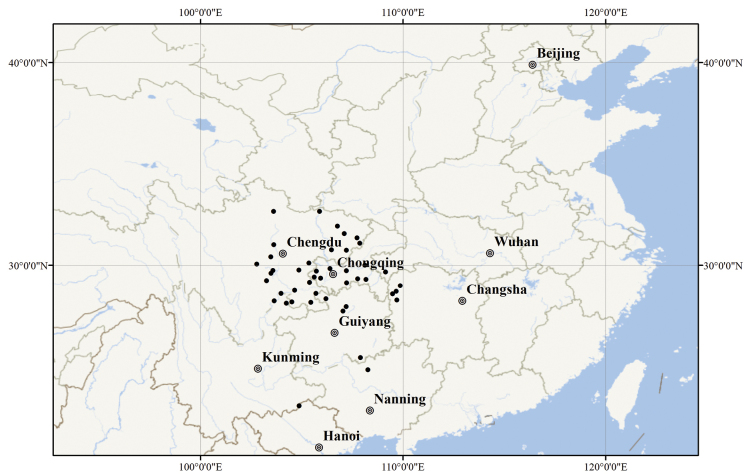
Distribution of *Saniculacaerulescens* (black circle).

*Saniculacaerulescens* is widely distributed in China (Chongqing, northern Guangxi, Guizhou, Hubei, western Hunan, Sichuan and Yunnan) and Vietnam (Hà Giang) (Fig. 10).

##### Habitat.

It grows on mountain slopes under forest or along ravine streams or at forest margins at elevations of 400–1700 m above sea level.

##### Phenology.

Flowering and fruiting from March to July.

##### Etymology.

The epithet *caerulescens* is derived from the Latin term referring to the blue hue of the petals in the flowers.

##### Additional specimens examined.

China. **Chongqing Municipality** • Beibei District, 25 March 1934, *T.T. Yu 2835* (NAS, PE) • Beibei District, Jinyun Mountain, 5 July 1956, *Anonymous 326* (SM) • Beibei District, Jinyun Mountain, 5 May 1956, *M.L. Li s.n.* (SM) • Beibei District, Jinyun Mountain, 12 July 1983, *M.L. Sheh 8367*4 (NAS) • Beibei District, Jinyun Mountain, alt. 480 m, 29 March 1964, *Sichuan Exped. 0060* (CDBI, PEY) • Beibei District, Jinyun Mountain, alt. 700 m, 12 May 1943, *S.J. Wang 1059* (NAS, PE) • Beibei District, Jinyun Mountain, alt. 740 m, 21 April 1956, *Sichuan & Guizhou Exped. 114* (PE) • Beibei District, Jinyun Mountain, alt. 800 m, 28 March 2013, *Z.F. Xu & W. Qin G0013* (KUN) • Beibei District, Jinyun Mountain, April 1939, *R.H. Shan 1166* (NAS) • Beibei District, Jinyun Mountain, May 1949, *Y.W. Law 286* (NAS) • Beibei District, Jinyun Mountain, *R.H. Shan 1297* (NAS) • Beibei District, Jinyun Mountain, *S.J. Wang 1502* (PE) • Bishan District, 29 May 1978, *Bishan Exped. 0129* (SM) • Dazu County, Yulong Town, alt. 400 m, 14 June 1978, *Dazu Exped. 0240* (SM) • Fuling District, Lidu District, alt. 900 m, 10 April 1979, *Fuling Exped. 0002* (SM) • Nanchuan County,1932, *T.H. Tu 2848* (IBK, NAS) • Nanchuan County, 1955, *SM Exped. 2785* (SM) • Nanchuan County, 11 March 1957, *S.G. Tang 1722* (SM) • Nanchuan County, alt. 1300 m, 4 June 1962, *Anonymous 95748* (SM) • Nanchuan County, alt. 500 m, 5 April 1979, *Anonymous 0027* (SM) • Nanchuan County, alt. 820 m, 1 April 1957, *C.H. Hsiung & T.L. Chou 90051* (PE) • Nanchuan County, Bailuping, alt. 840 m, 17 April 1957, *K.F. Li 60545* (KUN, PE) • Nanchuan County, Bailuping, alt. 970 m, 12 April 1957, *K.F. Li 60437* (KUN, PE) • Nanchuan County, Dashi Village, alt. 500–550 m, 7 April 1979, *Anonymous 0081* (SM) • Nanchuan County, Honghegou, alt. 700 m, 1 July 1983, *M.L. Sheh 83653* (NAS) • Nanchuan County, Mount Jinfo, 8 June 1935, *S.P. Chang 123* (NAS) • Nanchuan County, Mount Jinfo, alt. 1200 m, 2 June 1935, *K.L. Chu 1053* (NAS, PE) • Nanchuan County, Mount Jinfo, Daheba, 29°4'42.39"N, 107°12'9.15"E, alt. 666–688 m, 9 May 2021, *H.M. Li, Y.S. Zhang & X. Zhang LHM1139* (NAS) • Nanchuan County, Mount Jinfo, Daheba, 29°5'46.81"N, 107°12'0.5"E, alt. 704 m, 8 May 2021, *H.M. Li, Y.S. Zhang & X. Zhang LHM1132* (NAS) • Nanchuan County, Mount Jinfo, Daheba, alt. 650 m, 28 June 1983, *M.L. Sheh 83645* (NAS) • Nanchuan County, Mount Jinfo, Daheba, alt. 690 m, 26 April 1962, *Anonymous 95548* (SM) • Nanchuan County, Mount Jinfo, Daheba, alt. 785 m, 20 March 1957, *K.F. Li 60107* (PE) • Nanchuan County, Mount Jinfo, Daheba, alt. 880 m, April 1932, *T.H. Tu 2775* (IBK, NAS, PE) • Nanchuan County, Mount Jinfo, Daheba, alt. 1000 m, 7 April 1957, *K.F. Li 60356* (KUN, PE) • Nanchuan County, Mount Jinfo, Daheba, alt. 1300 m, 4 June 1962, *Anonymous 95748* (SM) • Nanchuan County, Mount Jinfo, Daheba, alt. 1550 m, 30 June 1957, *K.F. Li 62459* (PE) • Nanchuan County, Nanping Town, alt. 1000 m, 1 July 1957, *C.H. Hsiung & B.Q. Li 95026* (NAS, SM) • Nanchuan County, Sanjianghonghegou, alt. 700 m, 1 July 1983, *M.L. Sheh 83654* (NAS) • Nanchuan County, Sanquan Town, 29°4'47.28"N, 107°12'6.48"E, alt. 661 m, 26 May 2020, *W. Zhou & H.M. Li LHM1016* (NAS) • Nanchuan County, Sanquan Town, alt. 640 m, 7 March 1996, *Z.Y. Liu 15276* (E, HAST, K, KUN, P, PE) • Pengshui County, Lianhe Village, alt. 430 m, 12 May 2000, *Anonymous 0133B* (SM) • Rongchang District, Shuanghe Town, 24 April 1979, *Anonymous 619* (SM) • Shizhu County, Huangshui District, alt. 1400 m, 18 May 1978, *W.H. Wang 289* (CDBI) • Shizhu County, Shiliu Village, 29°44'35.96"N, 108°16'9.2"E, alt. 1219 m, 6 May 2021, *H.M. Li., Y.S. Zhang & X. Zhang LHM1125* (NAS) • Shizhu County, Xituo Town, alt. 150 m, 5 April 1979, *Shizhu Exped. 0040* (SM) • Wulong County, Furong River, alt. 300 m, 27 April 2008, *Z.Y. Liu 182499* (PE) • Wulong County, Furong River, alt. 350 m, 25 April 2008, *Z.Y. Liu 182290* (PE) • Yongchuan District, Banqiao Town, alt. 350 m, 23 April 1978, *Yongchuan Exped. 52* (SM) • Yongchuan District, Jiulong Village, alt. 500 m, 18 May 1978, *Yongchuan Exped. 248* (SM). **Guangxi Province** • Huanjiang County, Mulun National Nature Reserve, 107°58'41.85"N, 25°8'7.43"E, alt. 502 m, 15 May 2013, *Huanjiang Exped. 451226130315013LY* (GXMG, IBK) • Huangjiang County, Mulun National Nature Reserve, alt. 570 m, 16 April 2012, *R.C. Peng & L.F. Fu ML0955* (IBK) • Huangjiang County, Mulun National Nature Reserve, alt. 670 m, 19 April 2012, *Y.S. Huang, Y.B. Liao & M.Q. Han Y1288* (IBK) • Huanjiang County, Mulun National Nature Reserve, alt. 750 m, 28 February 2011, *W.B. Xu & L. Wu 11095* (IBK). **Guizhou Province** • Chishui City, 29 April 1965, *Guizhou Exped. 125* (HGAS) • Chishui City, Jinshagou, 20 May 2019, *L. Liu 201905203* (GNUG) • Libo County, Maolan Scenic Spot, alt. 340 m, 24 April 2018, *T. Jiang 20181675* (QNUN) • Libo County, Maolan Scenic Spot, alt. 420 m, 23 April 2018, *Z.H. Li 201814397* (QNUN) • Libo County, Maolan Scenic Spot, alt. 459 m, 19 April 2018, *F. Li 20185229* (QNUN) • Libo County, Maolan Scenic Spot, alt. 475 m, 17 April 2018, *X.J. Ma 20188141* (QNUN) • Libo County, Maolan Scenic Spot, alt. 493 m, 20 April 2018, *H.T. Long 20185417* (QNUN) • Libo County, Maolan Scenic Spot, alt. 498 m, 21 April 2018, *M.J. He 20185370* (QNUN) • Libo County, Maolan Scenic Spot, alt. 567 m, 21 April 2018, *K.Q. Zhang 20184413* (QNUN) • Suiyang County, Kuankuoshui, 12 May 2008, *S.Q. Zhang S4073* (ZY) • Suiyang County, Yinjianggou, 22 May 2014, *D. Hu XS14054478* (ZY) • Suiyang County, alt. 1050 m, 5 April 1990, *K.M. Lan 90–0006* (GZAC) • Suiyang County, 20 May 1986, *Q.S. Xie 201* (ZY) • Xishui City, Shaguangou, alt. 1055 m, 21 May 2015, *Q.Q. Ran SCH1503252* (ZY) • Xishui City, Shaguangou, alt. 978 m, 20 May 2015, *T. Xu SCH1503270* (ZY) • Xishui City, Shaguangou, alt. 987 m, 20 May 2015, *Y.L. Peng SCH1503246* (ZY) • Xishui City, Shaguangou, 15 May 2012, *Q. Li SCH1202116* (ZY) • Xishui City, Yinjianggou, alt. 978 m, 19 May 2015, *W.Y. Wu SCH1503057* (ZY) • Xishui City, Yong’an Town, 106°24'49.23"N, 28°12'16.12"E, alt. 1236.7 m, 29 March 2014, *M.C. Wang GYZ1403290916* (GYBG) • Zunyi City, Hejiagou, alt. 910 m, 20 May 2014, *M. Xie XS14054040* (ZY) • Zunyi City, Lengshui River, 5 March 2013, *J.H. Liu LSH031* (ZY) • Zunyi City, Shanpen District, Dingcun Village, 4 April 1959, *N Guizhou Exped. 0022* (HGAS, IBK). **Hubei Province** • Enshi City, Pingbaying National Forest Park, 108°57'46.67"N, 29°22'55.32"E, alt. 1404 m, 21 May 2021, *Y.S. Zhang LHM1120* (NAS). **Hunan Province** • Baojing County, Baiyunshan, 18 July 2009, *D.G. Zhang 080308007* (JIU) • Huayuan County, Gumiao River, alt. 200 m, 16 April 2008, *Y.G. Qu 080416062* (JIU) • Jishou City, Dehang Scenic Spot, 6 June 1993, *G.X. Chen 574* (JIU) • Yongshun County, Qingping Village, Dongba River, 110°11'10.7"N, 29°02'13.7"E, alt. 332 m, 23 March 2015, *K.D. Lei 4331271503231562* (JIU). **Sichuan Province** • Anyue County, Kongque Village, alt. 490 m, 10 April 1964, *Anonymous 366* (CDBI) • Anyue County, Shiyang Town, 6 May 1978, *Anonymous 1299* (SM) • Bazhong City, Bazhou District Zaolin Town, alt. 420 m, 14 April 1979, *Bazhong Exped. 1004* (SM) • Dazhou District, Xuanhan County, alt. 850 m, 6 May 1978, *Xuanhan Exped. 0167* (SM) • Dazhu County, Jixing Village, alt. 850 m, 16 May 1978, *Dazu Exped. 0125* (SM) • Dujiangyan City, Lingyan Mountain, 6 June 1959, *S. Jiang & C.L. Jin 549* (KUN) • Dujiangyan City, alt. 700 m, 17 April 1997, *D.Z. Lu 199704* (BJFC) • Ebian Yi autonomous county, 25 May 1959, *C.H. Hsiung & B.Q. Li 95026* (SM) • Emei Shan city,1939, G. Zhang 1907 (NAS) • Emei Shan City, 103°17'30.55"N, 29°35'12.17"E, alt. 1344.73 m, 2 June 2024, *C.F. Song, H.M. Li & J.W. Zhu LHM1557* (NAS) • Emei Shan city, Dacheng Temple Qingyin Pavilion, alt. 900 m, 27 March 1959, *Sichuan Econ. Plant Exped. 0209* (NAS, PE, SM) • Emei Shan City, Ereshan, 10 March 1949, *Chow & Hsie 784* (NAS) • Emei Shan City, Shuangfu Town, Yuelian Village, 18 May 1978, *Emei Exped. 122* (SM) • Emei Shan City, 1 April 1942, *W.P. Fang 18263* (TIE) • Emei Shan City, 27 March 1959, *Anonymous 404* (PE, SM) • Emei Shan City, *Sichuan Univ. Biol. Depart. Exped. 53902* (WUK) • Guang’an City, Jincheng Mountain, alt. 570 m, 29 April 1959, *Anonymous 00101* (SM) • Guangyuan City, Chaotian District, alt. 1750 m, 18 September 1972, *NE Yunnan Exped. 769* (KUN) • Hongya County, Wawu Mountain, 23 July 1950, *C.W. Yao 2440* (NAS, PE) • Jiajiang County, Huatou District, Menkanlin, 11 May 1959, *Sichuan Econ. Plant Exped. 8015* (PE, SM) • Junlian County, Haoba Town, Fujiagou, alt. 1200 m, 10 July 1977, *Anonymous 0539* (SM) • Kaijiang County, Nandinggou, alt. 1050–1100 m, 18 June 1978, *Kaijiang Exped. 0248* (SM) • Lu County, 10 May 1959, *Anonymous 5155* (SM) • Pingchang county, alt. 400 m, 15 June 1978, *Pingchang Exped. 384* (SM) • Qionglai City, alt. 800 m, 6 April 1979, *Anonymous 0079* (SM) • Songpan County, Huaying Mountain Huanglong Temple, 14 March 1941, *Y.C. Yong 4090* (NAS, PE) • Tianquan County, Yongxing District, alt. 910 m, 1 June 1982, *D.Y. Peng 45446* (CDBI) • Xuyong County, Shuiwei Town, Huagaoxi National Nature Reserve, 105°32'8"N, 28°16'36"E, alt. 419 m, 12 March 2013, *X.F. Gao, Z.M. Zhu & X.L. Zhao HGX11087* (CDBI) • Xuyong County, Shuiwei Town, Huagaoxi National Nature Reserve, alt. 1050–1150 m, 15 April 2012, *X.F. Gao, Z.M. Zhu & X.L. Zhao HGX10084* (CDBI) • Yibin City, Changning County, Wanling Town, alt. 560 m, 12 June 1977, *Anonymous 0044* (SM) • Zizhong County, Xinqiao Town, alt. 600 m, 28 March 1979, *Zizhong Exped. 86* (SM). **Yunnan Province** • de kan-tse-pin, *E.E. Maire s.n.* (P) • Long-ki,1894, *M. Delavay 4912* (P) • Long-ki,1894, *M. Delavay 4934* (P) • Long-ki, 1894, *M. Delavay s.n* (P, US) • Long-ki, 22 March 1894, *M. Delavay s.n.* (PE) • Long-ki, May 1894, *M. Delavay 4976* (P) • Zhaotong City, Shuifu County, Chenfengshan, 20 May 1901, *F. Ducloux 2130* (P) • Zhaotong City, Suijiang County, alt. 1307 m, 27 March 2023, *C.F. Song, H.M. Li & J.W. Zhu SCF0097* (NAS) • Zhaotong City, Suijiang County, alt. 1700 m, 4 July 1973, *Anonymous s.n* (HITBC) • Zhaotong City, Yongshan County, alt. 1513 m, 27 March 2023, *C.F. Song, H.M. Li & J.W. Zhu SCF0101* (NAS) • Zhaotong City, Yongshan County, alt. 1538 m, 27 March 2023, *C.F. Song, H.M. Li & J.W. Zhu SCF0103* (NAS).

Vietnam. **Hà Giang Province** • Quan Ba District, Tung Vai Commune, Thang Village, 104°51'48.8"N, 23°03'13.4"E, alt. 1050–1150 m, 21 April 2018, *L. Averyanov et al. VR 546* (LE) • Quan Ba District, Tung Vai Commune, Thang Village, 104°50'41.6"N, 23°03'41.5"E, alt. 1200–1400 m, 22 April 2018, *L. Averyanov et al. VR 629* (LE).

##### Note.

*Saniculacaerulescens* has been recorded in Chongqing, Guizhou, western Hunan, Sichuan and north-eastern Yunnan in China, as well as Hà Giang in Vietnam (Diels 1901; Boissieu 1906; Wolff 1913; Shan and Constance 1951; Hiroe 1979; Liou 1979; Wu 1984; Yang 1989; Liu 1997; Sheh and Phillippe 2005; Sun 2010). Pimenov (2017) also noted its presence in Hubei Province, although no specific specimens were cited. During our fieldwork in 2021, we were delighted to discover a population [*Y.S. Zhang LHM1120* (NAS)] in Enshi, Hubei. Furthermore, a critical examination of specimens under *Sanicula* L. confirmed that *S.caerulescens* is also distributed in northern Guangxi, as well as in all adjacent regions. Therefore, we clarify that the distribution of *S.caerulescens* extends across China (Chongqing, northern Guangxi, Guizhou, Hubei, western Hunan, Sichuan and Yunnan) and Vietnam (Hà Giang).

## Supplementary Material

XML Treatment for Sanicula caerulescens

## ﻿Additional information

### Author ORCIDs

Hui-min Li https://orcid.org/0000-0001-6660-7620

Wei Zhou https://orcid.org/0000-0003-1627-1529

Jun-wen Zhu https://orcid.org/0009-0000-5554-445X

Chun-feng Song https://orcid.org/0000-0003-2077-0486

### Data availability

All of the data that support the findings of this study are available in the main text.
